# Comparison of Access to Novel Drugs for Lymphoma and Chronic Lymphocytic Leukemia Between India and the United States

**DOI:** 10.1200/GO.20.00012

**Published:** 2020-07-21

**Authors:** Vishwanath Sathyanarayanan, Christopher R. Flowers, Swaminathan P. Iyer

**Affiliations:** ^1^ Department of Medical Oncology, Apollo Hospitals, Bangalore, India; ^2^ Department of Lymphoma and Myeloma, The University of Texas MD Anderson Cancer Center, Houston, TX

## Abstract

This review will compare and contrast the costs and access to novel drugs for treating chronic lymphocytic leukemia (CLL) and lymphoma in the United States and India during the last 5 years. Clinical outcomes for patients with hematologic malignancies have improved significantly since the approval of immunotherapeutic and targeted therapies. These new treatments have had an impact on overall outcomes and have helped determine the design for translational research and future trials. Although most of these novel drugs called “innovators” are initially approved and marketed in the United States, several have also become available in countries such as India. With the expiration of patents, generic versions of innovator drugs have increased and accessibility has improved for patients. The advent of biosimilars is another route for expanding access to biologic compounds. As a result, the development costs for developing these drugs are lower, and consequently, the costs for the patient are often lower. Although the delivery of cancer care is not the same in India as it is in the United States, the introduction of biosimilars and generics has helped bridge the gap. This has made treatment of CLL and lymphoma similar in both countries and has had the same impact on patient outcomes and quality of life. Compulsory licensing for essential medications, as stipulated by the Doha Declaration, and capping of drug prices could improve global access to treatments for CLL and lymphoma.

## INTRODUCTION

Blood cancers are a significant public health problem worldwide and a leading cause of death in the United States and India. According to GLOBOCAN 2018, the annual incidence of lymphoma is 82,548 in the United States and 37,225 in India.^1^ The annual incidence of chronic lymphocytic leukemia (CLL) was 50,149 in the United States and 42,055 in India.^2^ Over the past 5 years, the US Food and Drug Administration (FDA) has approved 24 new indications in lymphoma and 11 in CLL (Table 1).^3^ Most clinical trials for these novel drugs are conducted in the United States, and the drugs are available for use soon after, but only in the United States. The approval process for new drugs in India is managed by the Central Drugs Standard Control Organisation and generally lags behind approval in the United States by at least 2 years.^4^ In addition, a decade can elapse before generics and biosimilars reach clinical practice. Our study aims to increase understanding of the similarities and differences regarding cancer care delivery, accessibility, cost, and the potential impact on survival for patients in the United States and India with the advent of these novel drugs for lymphoma and CLL.

**CONTEXT**

**Key** **Objective**
Blood cancers are a leading health problem in the United States and India. We compare the cost of and access to novel drugs for treating chronic lymphocytic leukemia (CLL) and lymphoma between the United States and India during the last 5 years.
**Knowledge Generated**
Delivery of cancer care in the United States is different from that in India. In the United States, around 90% of the population has health insurance. In India, a majority of the population pays for medical expenses out of pocket. The cost of drug development is high, and most novel drugs are initially developed and marketed in the United States, but it takes several years before the drugs become available in India. The development of biosimilars has increased access to and affordability of biologics for the treatment of CLL and lymphoma in India.
**Relevance**
The development of biosimilars has increased access to and affordability of biologics for the treatment of CLL and lymphoma in India. The overall outcome and quality of life is rather similar in the two countries with the advent of biosimilars and generics. Future strategies to ensure universal access include expanding the availability of biosimilars, capping drug prices, expanding insurance coverage, and constructing a hub-and-spoke rural outreach model to make novel drugs accessible to all patients.

**TABLE 1:**
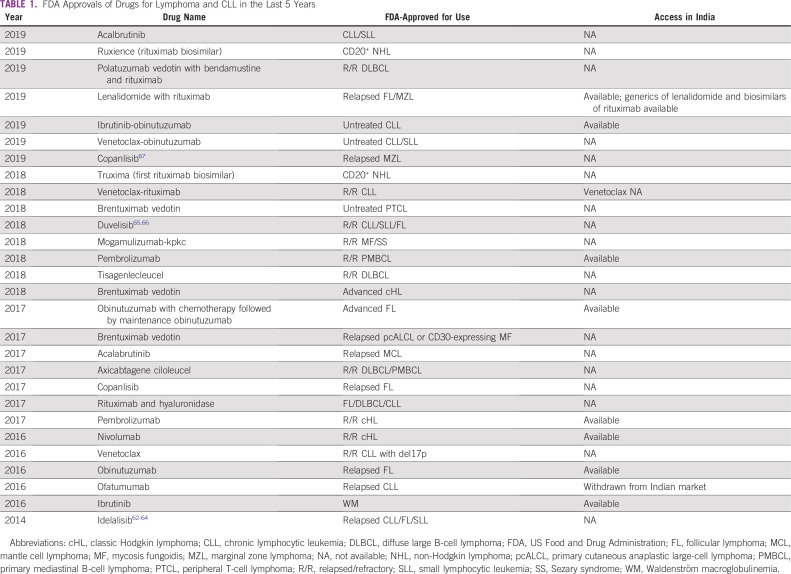
FDA Approvals of Drugs for Lymphoma and CLL in the Last 5 Years

## DELIVERY OF CANCER CARE

The cancer care delivery systems in the United States and India illustrate the differences between health care in a developed and in a developing nation. The United States spends 17.8% of its gross domestic product on health care and often is the first country to adopt new therapies.^5^ Most novel drugs recently approved for treating cancer are priced at more than $100,000 per year and are thus affordable only with health insurance.^6-9^ Approximately 90% of the US population is covered by health insurance.^10^ In addition, the Patient Protection and Affordable Care Act (2010) has expanded insurance coverage to include cancer care.^11^ Individuals with lymphoma who live in rural areas and those who are uninsured have inferior outcomes probably because they have less access to cancer care.^12-16^

Cancer care delivery in India is the opposite. India spends around 3.89% of its gross domestic product on health care.^17^ The low patient:oncologist ratio in India (1:2,000) adds to the increasing demands of cancer care.^18^ Most people pay out of pocket for medical expenses, and only a minority have health insurance.^19,20^ A substantial portion of the population lives in rural areas with little or no awareness of what health care options are available, and they have limited resources to pay for specialized health care. We have not captured comparisons between countries in terms of rural accessibility. The regional cancer centers in India provide health care at a subsidized rate only to a limited population.^21^ The National Cancer Grid is a recent initiative by the Government of India under the Ayushman Bharat-Pradhan Mantri Jan Arogya Yojana, which extends annual financial support up to $7,000 for patients with cancer.^22^

## DRUG PRICING IN THE UNITED STATES

The average cost of new drug development is approximately $100 million, and the projected cost for a successful approval is approximately $1 billion, so the prices for marketed drugs are higher. Generic medicines and the biosimilars that enter the system later can help to reduce the cost of the drugs for the consumer.^23,24^ The Drug Price Competition and Patent Term Restoration Act (Hatch-Waxman Act, 1984) has led to accelerated methods for approving generic drugs.^25^ However, in some cases, prices for cancer drugs are kept high by pharmaceutical company lobbying, which can also potentially delay the entry of generics into the market.^26^ The benchmark for drug reimbursement is set by Centers for Medicare & Medicaid Services. Effective January 1, 2017, payment for infusion drugs is based on Section 1847A of the Social Security Act and implies that most of the payments will be based on the average sales price of these drugs.^27-29^ Accordingly, Medicare pays 106% of the average sales price for most drugs covered under Part B.

## BIOSIMILARS AND GENERICS

Once the patents for the innovative drug expire after a stipulated period, a window of opportunity opens for producing a similar product called a biosimilar at a much lower cost without compromising efficacy.^30^ To date, the number of these agents that receive approval in the United States has been limited. Relevant agents include the pegfilgrastim biosimilars and the two biosimilars of rituximab.^31,32^ Because of a thriving pharmaceutical industry, the generics and biosimilars are more affordable in India. The first biosimilar in India was approved in 2007, and it reached a market value of $2.2 billion in 2017.^33^ Table 2 lists cost information for drugs in the United States and India for standard therapies for lymphomas and CLL. The costs appear to be different because of the method used to convert local currencies into US dollars. The World Bank classifies India as a low- and middle-income country. Although the current prices for drugs have been converted by using foreign exchange rates, the prices can be different if the conversion uses purchasing power parity.^34^ To illustrate, there are 70.84 Indian rupees to 1 US dollar using foreign exchange rates, but only 18.128 rupees to 1 US dollar using purchasing power parity.

**TABLE 2:**
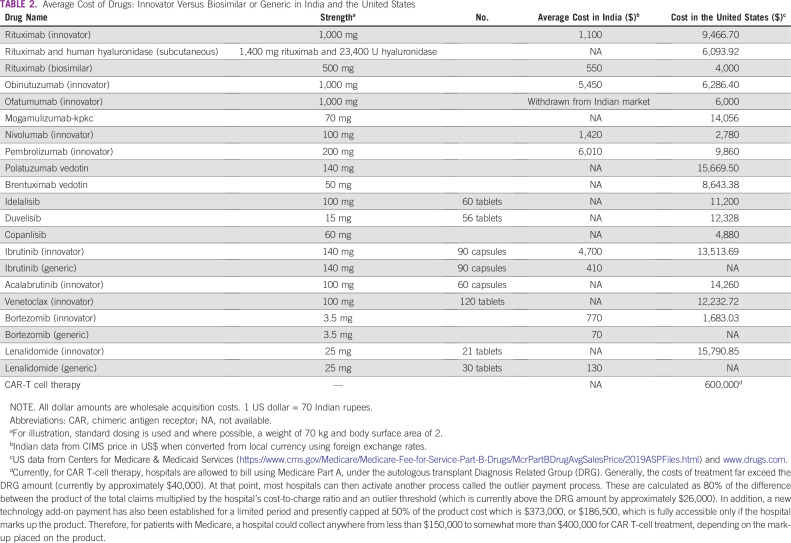
Average Cost of Drugs: Innovator Versus Biosimilar or Generic in India and the United States

## NOVEL DRUGS FOR LYMPHOMA AND CLL

Several novel drugs have shown promising results and have improved patient outcome in CLL and lymphoma. This section describes the FDA approvals and access to novel drugs in the United States and India during the last 5 years. Table 1 summarizes the new drug approvals and Table 2 provides an average cost of these agents in the United States and India.

### Antibodies

#### Rituximab.

Rituximab is an anti-CD20 monoclonal antibody approved in the United States for treating B-cell non-Hodgkin lymphoma. It is also approved for use in untreated and relapsed CLL, as an induction and maintenance therapy in follicular lymphoma (FL), and in combination with ibrutinib for Waldenström macroglobulinemia.^35,36^ Recent approvals include rituximab combined with lenalidomide for treating relapsed FL and marginal zone lymphoma (MZL) and rituximab combined with venetoclax for treating relapsed CLL.^3^ Truxima (rituximab-abbs) and Ruxience (rituximab-pvvr) are rituximab biosimilars recently approved by the FDA in the United States that cost less than rituximab.^31,32^

The use of anti-CD20 antibody in India is quite similar to the way it is used in the United States. The innovative drug rituximab has been available since the early 2000s at a higher price; when the biosimilar Reditux became available in 2007, the price dropped and the drug was made available across the country.^37,38^ In a retrospective study of 173 patients with diffuse large B-cell lymphoma (DLBCL), overall response rate (ORR), toxicity, progression-free survival (PFS), and overall survival (OS) were similar between Reditux and rituximab.^39^ The pharmacokinetic and toxicity profiles in 21 patients who received Reditux (biosimilar) were found to be comparable to those of patients receiving rituximab.^40^ With an effective patient assistance program, the access to rituximab biosimilars has improved in India. The subcutaneous formulation of rituximab, which was approved for treating FL, is not available in India.^41,42^

#### Obinutuzumab.

The fully humanized anti-CD20 antibody obinutuzumab was initially approved for treating relapsed CLL in 2013 and was subsequently approved as part of induction followed by maintenance in untreated advanced FL on the basis of results from the Gallium trial (ClinicalTrials.gov identifier: NCT013322968). Patients were randomly assigned to obinutuzumab plus chlorambucil chemotherapy or rituximab plus chlorambucil followed by obinutuzumab or rituximab maintenance in responders. The estimated 3-year PFS rate was 80.0% versus 73.3% (hazard ratio [HR], 0.66; *P* = .001) favoring the obinutuzumab arm. Adverse events (AEs) of grade 3 or higher were seen more often in the obinutuzumab group (74.6% *v* 67.8%).^43^ Other additional indications included ibrutinib-obinutuzumab and venetoclax-obinutuzmab for untreated CLL.^44,45^ Obinutuzumab is currently manufactured and marketed by Genentech in the United States. In India, it is used sparingly used because of its high cost.

#### Ofatumumab.

This second-generation CD20 antibody^46^ was initially available in India but was withdrawn from commercial use in 2018.^47^

#### Mogamulizumab-kpkc.

Mogamulizumab is a humanized monoclonal antibody against the chemokine receptor CCR-4 and was approved for adult patients with relapsed or refractory (R/R) mycosis fungoides or Sezary syndrome. In the phase III MAVORIC trial (ClinicalTrials.gov identifier: NCT01728805), patients on the mogamulizumab arm had a median PFS of 7.7 months versus 3.1 months for those on the vorinostat arm (HR, 0.53; *P* < .001); 41% had grade 3 or 4 AEs.^48^ This drug is currently not available in India.

#### Nivolumab and pembrolizumab.

Nivolumab is currently FDA approved for adult patients with R/R classic Hodgkin lymphoma (cHL) with previous autologous stem cell transplantation (ASCT). In 95 patients with relapsed cHL after ASCT and brentuximab vedotin, nivolumab produced a 65% ORR, and the median duration of response was 8.7 months. Serious AEs were reported in 21% of patients.^49^ Pembrolizumab is approved for treating refractory cHL and was recently granted accelerated approval for treating refractory primary mediastinal large B-cell lymphoma. The KEYNOTE-170 trial (ClinicalTrials.gov identifier: NCT02576990) was a multicentre, single-arm trial in 53 patients with R/R primary mediastinal large B-cell lymphoma and had an ORR of 45%. Although 61% had AEs, they were mostly grade 1 to 2 and were manageable.^50,51^ Nivolumab and pembrolizumab are available in India and are accessible to patients at an average cost of approximately $1,420 (100 mg) and $6,010 (200 mg), respectively.^52-54^

### Antibody-Drug Conjugates

#### Polatuzumab vedotin.

The FDA recently approved the novel CD79b-directed antibody-drug conjugate polatuzumab vedotin in combination with bendamustine and rituximab for adult patients with refractory DLBCL who had disease progression after 2 lines of chemotherapy. In a multicenter clinical trial (ClinicalTrials.gov identifier: NCT02257567) of 80 patients with relapsed DLBCL, the complete response (CR) rates and the ORR was higher with the polatuzumab vedotin plus benadmustine and rituximab as compared to the control arm of bendmustine plus rituximab (40% vs 18%; 63% vs 25% respectively). The combination had higher grade 3 to 4 cytopenias.^55^ Currently, polatuzumab is unavailable in India.^56^

#### Brentuximab vedotin.

Brentuximab vedotin was initially approved in 2011 for patients with relapsed Hodgkin lymphoma whose disease had progressed after ASCT or after 2 previous chemotherapy treatments and who were ineligible for a transplantation; an additional indication was added for patients with relapsed anaplastic large-cell lymphoma.^57^ In March 2018, brentuximab vedotin was approved by the FDA for treatment-naive advanced cHL on the basis of the ECHELON-1 trial (ClinicalTrials.gov identifier: NCT01712490). In all, 1,334 patients were randomly assigned to receive either brentuximab vedotin plus doxorubicin, vinblastine, and dacarbazine (AVD) or bleomycin plus AVD. There was a 23% reduction in the risk of a PFS event in the brentuximab vedotin arm. Occurrence of neutropenia and peripheral neuropathy were higher in the brentuximab vedotin arm (58% *v* 45% and 67% *v* 43%, respectively) as compared with AVD plus bleomycin.^58^

In 2018, the FDA also approved brentuximab vedotin for the treatment of untreated systemic anaplastic large-cell lymphoma (ALCL) or other CD30^+^ T-cell lymphomas (TCLs) with cyclophosphamide, doxorubicin, and prednisone (CHP). The phase III ECHELON-2 trial (ClinicalTrials.gov identifier: NCT01777152) was a randomized study of brentuximab vedotin plus CHP versus cyclophosphamide, doxorubicin, vincristine, and prednisone (CHOP) in 452 patients with newly diagnosed CD30^+^ TCL. The median PFS was 48.2 months with brentuximab vedotin plus CHP and 20.8 months with CHOP, with an HR of 0.71; there was improvement in OS (HR, 0.66). AEs were similar in both arms.^59^ Brentuximab vedotin is currently not available in India; TCL is treated with CHOP with or without etoposide followed by ASCT.^60^ R/R cHL is treated with immune checkpoint inhibitors or salvage chemotherapy in India, and occasionally brentuximab vedotin is imported and incorporated into some treatment regimens.^61^

### BTK Inhibitors and PI3 Kinase Inhibitors

#### Ibrutinib.

The Bruton tyrosine kinase (BTK) inhibitor ibrutinib is FDA approved for relapsed and untreated CLL, relapsed mantle cell lymphoma (MCL), relapsed MZL, and Waldenström macroglobulinemia.^62-67^ In the phase III trial (ClinicalTrials.gov identifier: NCT01886872) of ibrutinib versus ibrutinib with rituximab or bendamustine and rituximab in 547 elderly patients with untreated CLL, the median PFS was higher with patients receiving ibrutinib alone or with rituximab. The grade 3 to 4 hematologic AEs were greater with bendamustine and rituximab (61%) than with ibrutinib (41%) or with ibrutinib plus rituximab (39%).^68^ In the extended follow up of the RESONATE 2 trial (Clinical Trials.gov identifier: NCT01722487), the overall response rate was 92% with ibrutinib. At a median follow-up of 60 months, the PFS and OS for ibrutinib versus chlorambucil was-PFS estimates at 5 years: 70% vs 12%; HR: 0.146; 95% CI, 0.098 to 0.218; OS estimates at 5 years: 83% vs 68%; HR: 0.450; 95% CI, 0.266 to 0.761). There was a 88% reduction in risk of progression or death in the ibrutinib arm (P <0.001). Grade 3/4 AEs decreased over time except for atrial fibrillation.^63,69^ In 197 patients treated with ibrutinib, similar ORR, PFS, and OS were noted in 37 patients (19%) receiving a reduced median dose of 4.3 mg/kg per day compared with standard doses.^70^ A smaller Indian study also reported a similar efficacy with low-dose ibrutinib.^71^

Ibrutinib has also been approved for treating R/R MZL on the basis of a phase II trial in 63 patients who had an ORR of 46%. The median time to initial response was 4.5 months and PFS was 14 months.^65^ Ibrutinib is available for patients in India, but the cost remains high at this time ($4,700 for 90 capsules [140 mg]). Recently, a generic formulation has become available in India at $410 for 90 capsules (140 mg).^72^

#### Acalabrutinib.

Acalabrutinib is a second-generation BTK inhibitor approved by the FDA for treating patients with refractory MCL on the basis of the ACE LY-004 trial (ClinicalTrials.gov identifier: NCT02213926). The ORR was 81%, and the median duration of response was not reached, with a median follow-up of 15.2 months. Grade 3 or worse AEs were neutropenia in 10%, anemia in 9%, and pneumonia in 5%; there was 1 instance of hemorrhage.^73^ The impressive PFS results in patients with relapsed CLL, including those with high-risk cytogenetics in the phase III ASCEND trial (ClinicalTrials.gov identifier: NCT02970318), led to approval for this indication.^3,74^ In combination with obinutuzumab (ClinicalTrials.gov identifier: NCT02475681), there was an improvement in PFS compared with obinutuzumab-chlorambucil in more than 500 patients with previously untreated CLL.^3,75^ Acalabrutinib is not available in India. Patients with R/R MCL are being treated with ibrutinib or lenalidomide with rituximab; patients eligible for ASCT undergo induction with salvage chemotherapies followed by ASCT. Although the outcomes may be similar, a single-agent therapy would be more convenient and less toxic for patients compared with chemotherapy with ASCT or lenalidomide with rituximab. In addition, the recent availability of the generic ibrutinib in India at an affordable price may delay entry of acalabrutinib to India.

The approved PI3 kinase inhibitors, such as idelalisib,^76-78^ duvelisib,^79,80^ and copanlisib,^81^ are not available in India. Because of the increased number of AEs compared with those with BTK inhibitors in CLL and lenalidomide in indolent lymphomas, this lack of access has no real impact.

### BCL-2 Inhibitor

Venetoclax is an oral BCL-2 inhibitor that received FDA approval for treating patients with relapsed CLL who had a 17p deletion.^82^ Subsequently, on the basis of the MURANO study (ClinicalTrials.gov identifier: NCT02005471), venetoclax with rituximab was approved for treating patients with CLL or small lymphocytic lymphoma who had received at least 1 previous therapy. The comparison arm was bendamustine plus rituximab. Tumor lysis syndrome and neutropenia were higher with venetoclax.^83^ The combination of venetoclax-obinutuzumab in a trial of 432 elderly patients with untreated CLL also has been approved, and it showed a benefit in the high-risk group (ClinicalTrials.gov identifier: NCT02242942). The 2-year PFS was higher in the venetoclax-obinutuzumab arm (88.2% *v* 64.1%, with 28 months of follow-up) as compared with chlorambucil plus obinutuzumab. The combination was well tolerated and it had similar rates of neutropenia and infections as the control arm of chlorambucil plus obinutuzumab.^45^ Patients in India currently do not have access to venetoclax.

### Proteosome Inhibitor

Bortezomib is a protease inhibitor approved for patients with relapsed MCL and subsequently for treating patients with untreated MCL who are ineligible for ASCT.^84,85^ The generic version is affordable and is mainly used for managing multiple myeloma. It has not had an impact on care for patients with lymphoma in India because lenalidomide, rituximab, and ibrutinib are used to treat patients with relapsed MCL.

### Immunomodulators

#### Lenalidomide.

Lenalidomide is an immunomodulatory agent approved for patients with relapsed MCL.^86^ It has been approved in the United States for R/R FL or MZL, based on the AUGMENT randomized clinical trial (ClinicalTrials.gov identifier: NCT01472562). The lenalidomide-rituximab arm had a median PFS of 39 months versus 14 months for rituximab alone. A higher percentage of infections (63% *v* 49%), neutropenia (58% *v* 23%), and rash (32% *v* 12%) was seen in the combination arm, but overall the AEs were tolerable in both arms.^87^ The data from several studies show an improvement in PFS in patients with relapsed lymphomas and CLL and also in elderly patients with DLBCL who received lenalidomide as maintenance therapy after rituximab plus CHOP (R-CHOP).^88-91^ Because this drug is available as an affordable generic, access to this novel drug has expanded significantly in India.

#### CAR T-cell therapy.

In October 2017, the FDA approved axicabtagene ciloleucel for patients with R/R DLBCL on the basis of a single-arm multicenter trial of 111 adult patients (ZUMA-1; ClinicalTrials.gov identifier: NCT02348216). The ORR was 72% and the CR rate was 51%. The responses were sustained, and the median OS was not reached at 2 years, but an estimated 50.5% of patients were still alive at 24 months.^92,93^ In May 2018, the FDA approved tisagenlecleucel for adults with relapsed DLBCL after 2 lines of chemotherapy on the basis of a phase II single-arm study of 92 patients. The ORR was 50%, and the CR was 32%.^94^ Neutropenia, anemia, thrombocytopenia, and chimeric antigen receptor (CAR) T-cell–specific AEs such as cytokine release syndrome and neurologic toxicities were seen in both trials. Therapy using CAR T cells involves substantial cost, expertise, and infrastructure. CAR T-cell therapy may be the best treatment currently available for patients with relapsed lymphomas who otherwise have a dismal prognosis, but it is not available in India.^29,95,96^

### Possible Strategies for Improving the Accessibility of Drugs in the United States and India

Effective management of lymphoid cancers can often provide patients with long-term survival if they are treated appropriately with novel drugs. Thus, the role of compulsory licensing is worth considering. The Doha Declaration on mandatory licensing allowed member countries to circumvent patent rights so that patients would have access to essential medications. There are several hurdles in this path that need to be addressed. A step in the right direction would be to expand the market for biosimilars and generics in the United States and India. Furthermore, global trials for these drugs might enhance accessibility.^97,98^ In India, the Prime Minister’s health insurance scheme caps the drug costs to reduce the financial burden.^22^ Another step toward improving accessibility would be to provide universal insurance coverage. A systematic effort from the government agencies and use of a hub-and-spoke model for rural outreach may be required to improve access in remote areas of India. In the United States, by allowing Medicare and the FDA to have a say in drug prices, laws that enable importing drugs and negotiating drug prices can help reduce drug costs.^99^ Here, we have outlined the differences in availability and affordability of cancer drugs between India and the United States.

In conclusion, the advent of biosimilars has reduced the cost of and made treatment of CLL and lymphoma similar in the United States and India in terms of survival and patient quality of life. Innovative strategies would help expand access to other novel agents to patients in India as well. In addition, implementing the Doha Declaration, having compulsory licensing, capping drug prices, expanding insurance coverage, and making cancer care available to all people irrespective of their economic, social, racial, and geographical backgrounds would make treatment for cancer globally accessible.
